# The Cramér–Rao Bounds and Sensor Selection for Nonlinear Systems with Uncertain Observations

**DOI:** 10.3390/s18041103

**Published:** 2018-04-05

**Authors:** Zhiguo Wang, Xiaojing Shen, Ping Wang, Yunmin Zhu

**Affiliations:** School of Mathematics, Sichuan University, Chengdu 610064, China;wangzg315@126.com (Z.W.); yidianyuanzhidian@163.com (P.W.); ymzhu@scu.edu.cn (Y.Z.)

**Keywords:** Cramér–Rao bound, sensor selection, uncertain measurement, target tracking

## Abstract

This paper considers the problems of the posterior Cramér–Rao bound and sensor selection for multi-sensor nonlinear systems with uncertain observations. In order to effectively overcome the difficulties caused by uncertainty, we investigate two methods to derive the posterior Cramér–Rao bound. The first method is based on the recursive formula of the Cramér–Rao bound and the Gaussian mixture model. Nevertheless, it needs to compute a complex integral based on the joint probability density function of the sensor measurements and the target state. The computation burden of this method is relatively high, especially in large sensor networks. Inspired by the idea of the expectation maximization algorithm, the second method is to introduce some 0–1 latent variables to deal with the Gaussian mixture model. Since the regular condition of the posterior Cramér–Rao bound is unsatisfied for the discrete uncertain system, we use some continuous variables to approximate the discrete latent variables. Then, a new Cramér–Rao bound can be achieved by a limiting process of the Cramér–Rao bound of the continuous system. It avoids the complex integral, which can reduce the computation burden. Based on the new posterior Cramér–Rao bound, the optimal solution of the sensor selection problem can be derived analytically. Thus, it can be used to deal with the sensor selection of a large-scale sensor networks. Two typical numerical examples verify the effectiveness of the proposed methods.

## 1. Introduction

In practical problems, we always encounter some sensors have the uncertain measurement subjected to random interference, natural interruptions or sensor failures. Using the mode parameters without considering the uncertainty is unavailable, and there are a lot of researchers that have studied the state estimation with uncertain measurement, such as [1,2,3,4]. In this paper, we consider the uncertainty caused by occlusions, i.e., the sensors may not be able to observe the target when blocked by some obstacles [5]. For the linear dynamic systems involving uncertainty in [6,7], the authors use a Kalman filter to track the target. However, it is difficult to obtain the optimal estimation for a nonlinear uncertain dynamic system, but we are particular interested in measuring their efficiency. For this purpose, it is natural to compare a lower bound of the estimation error, which gives an indication of performance limitations. Moreover, it can be used to determine whether imposed performance requirements are realistic or not.

The most popular lower bound is the well-known Cramér–Rao bound (CRB). In time-invariant statistical models, the estimated parameter vector is usually assumed to be real-valued (non-random). The lower bound is given by the inverse of the Fisher information matrix. When we deal with the time-varying systems, the estimated parameter vector is modeled randomly. A lower bound that is analogous to the CRB for random parameters is derived in [8], and this bound is also known as the Van Trees version of the CRB, or referred to as posterior CRB (PCRB). In fact, the underlying static random system needs to satisfy the regularity condition, which is absolute integrability of the first two derivatives of all related probability density functions. The first derivation of a sequential PCRB version applicable to discrete-time dynamic system filtering is done in [9] and then extended in [10,11,12]. The most general form of sequential PCRB for discrete-time nonlinear systems is presented in [13]. Together with the original static form of the CRB, these results serve as a basis for a large number of applications [14,15,16].

Most of the papers on PCRB are obtained without considering the uncertainty in the dynamic systems. When the sensors have uncertain measurements, we need to consider the influence of the uncertainty [17,18]. The CRB is presented in [19,20] to target tracking with detection probability smaller than one. If the uncertain measurement is prone to discretely-distributed faults, a Cramér–Rao-type bound is shown in [21]. Actually, the authors in [22,23] have considered uncertainty as the mixed Gaussian probabilistic model, where the sensor observation is assumed to contain only noise if the sensor cannot sense the target. Therefore, we hope to derive a recursive PCRB based on the uncertain model of the Gaussian mixture distribution.

Since the PCRB needs to compute the Fisher information, which is obtained by the derivatives of the log likelihood function of the Gaussian mixture model, and it is much more difficult than the case of a single Gaussian distribution. The reason is that the presence of the summation that occurs inside of the logarithm, and the PCRB of the Gaussian mixture model needs to compute the complex integral, which is with respect to the joint probability density function of the sensor measurements and the target state. These reasons motivate us to research another approach to derive the PCRB.

In large wireless sensor networks (WSNs), sensors are battery-powered devices with limited signal processing capabilities [24,25]. In such situations, it is inefficient to utilize all the sensors including the uninformative ones, which is hardly helpful to the tracking task but still consumes resources. This issue has been researched and shown via the development of sensor selection schemes, whose goal is to select the best non-redundant set of sensors for the tracking task while satisfying the resource constraints [26,27]. The previous research [28,29] on sensor selection assumes that the target tracking process does not have any interruptions. As the sensor observations are quite uncertain, we need to consider the sensor selection based on the proposed PCRB.

In this paper, we use two methods to derive the PCRB to effectively overcome the difficulties caused by uncertainty. The first method is based on the recursive formula of the Cramér–Rao bound and the Gaussian mixture model. Nevertheless, it needs to compute a complex integral based on the joint probability density function of the sensor measurements and the target state, which leads to the computation burden of this method being relatively high, especially in large sensor networks, so that it is not better using this PCRB as a measure criteria of the sensor selection. In order to reduce the computation burden and deal with the sensor selection of a large-scale sensor networks, our contributions are as follows:

- Inspired by the idea of the expectation maximization algorithm, we introduce some 0–1 latent variables to treat the Gaussian mixture model. Since the regular condition of the posterior Cramér–Rao bound is unsatisfied in the discrete uncertain system, we use some continuous variables to approximate the discrete latent variables, then a new Cramér–Rao bound can be achieved by a limiting process of the Cramér–Rao bound of the continuous system. The Cramér–Rao bound avoids the complex integral with a less computation burden.
- Based on the proposed posterior Cramér–Rao bound, the sensor selection problems for the nonlinear uncertain dynamic system can be efficiently solved, and the optimal solution of the sensor selection problem can be derived analytically. Thus, it can be used to deal with the sensor selection for the large-scale sensor networks.

The remainder of this paper is organized as follows. The system uncertain model is defined and the problem is formulated in Section 2. The PCRB for the dynamic system with uncertain observations is detailed and justified in Section 3. The optimal sensor selection with uncertain observations is shown in Section 4. Two numerical examples are presented in Section 5. Finally, the conclusions are offered in the final section.

## 2. Problem Formulation

Consider the *L*-sensor nonlinear dynamic systems with the uncertain observations [5,30],

(1) $$\begin{array}{ccc} x_{k} & = & f_{k}\left(x_{k-1}\right)+w_{k}, \end{array}$$

(2) $$\begin{array}{ccc} y_{k}^{i} & = & \left\{\begin{array}{cc} h_{k}^{i}\left(x_{k}\right)+v_{k}^{i} & \mathrm{with}\;\mathrm{probability}\;p_{k}^{i},\\ v_{k}^{i} & \mathrm{with}\;\mathrm{probability}\;1-p_{k}^{i}, \end{array}\right.\\ & & i=1,2,\ldots ,L, \end{array}$$

where $p_{k}^{i}$ is the sensing probability of sensor *i*, $x_{k}$ is the state of system at time *k*, $y_{k}^{i}$ is the measurement at the *i*th sensor, i=1,…,L, $f_{k}\left(x_{k-1}\right)$ is the nonlinear state function, and $h_{k}^{i}\left(x_{k}\right)$ is the nonlinear measurement function of $x_{k}$ at the *i*th sensor. $w_{k}$ and $v_{k}^{i}$ are the state noise and the measurement noise, respectively, and they are mutually independent. $v_{k}^{i}$ is assumed to be independent across time steps and across sensors. The measurement information of the *i*th sensor is denoted by $Y_{k}^{i}=\left\{y_{1}^{i},y_{2}^{i},\ldots ,y_{k}^{i}\right\}$.

Assume that $w_{k}$ and $v_{k}^{i}$ are white Gaussian noise with $N(0,Q_{k})$ and $N(0,R_{k}^{i})$, i=1,…,L, respectively, where, $Q_{k}$ and $R_{k}^{i}$ are the corresponding covariance matrices. We also assume that the initial state $x_{0}∼N\left({\hat{x}}_{0},\Sigma_{0}\right)$, and, if $x_{k}$ is given, then the measurement $y_{k}^{i}$ follows the Gaussian distribution $N(h_{k}^{i}\left(x_{k}\right),R_{k}^{i})$ with probability $p_{k}^{i}$, and follows the Gaussian distribution $N(0,R_{k}^{i})$ with probability $1-p_{k}^{i}$, i.e.,

(3) $$p\left(y_{k}^{i}|x_{k}\right)=p_{k}^{i}N\left(h_{k}^{i}\left(x_{k}\right),R_{k}^{i}\right)+\left(1-p_{k}^{i}\right)N\left(0,R_{k}^{i}\right).$$

Obviously, the conditional probability density function is a Gaussian mixture distribution, which ishard to calculate the PCRB. This difficult problem motivates us to introduce a hidden state variable, which draws lessons from the idea of the expectation maximization (EM) algorithm [31].

We introduce the 0–1 hidden state variables $I_{k}^{i},i=1,2,\ldots ,L$, which indicate whether the dynamic system has uncertainty. In other words, if $I_{k}^{i}=1$, then $y_{k}^{i}=h_{k}^{i}\left(x_{k}\right)+v_{k}^{i}$, and $I_{k}^{i}=0$, then $y_{k}^{i}=v_{k}^{i}$. Now, we transfer the nonlinear systems (1) and (2) as follows:

(4) $$\left\{\begin{array}{ccc} x_{k} & = & f_{k}\left(x_{k-1}\right)+w_{k},\\ I_{k}^{i} & = & 0\cdot I_{k-1}^{i}+{\tilde{w}}_{k}^{i}, \end{array}\right.$$

(5) $$y_{k}^{i}=I_{k}^{i}\cdot h_{k}^{i}\left(x_{k}\right)+v_{k}^{i},\;i=1,2,\ldots ,L.$$

Then, the compact form for Equations (4) and (5) can be written as follows:

(6) $${\overset{˘}{x}}_{k}=F_{k}\left({\overset{˘}{x}}_{k-1}\right)+W_{k},$$

(7) $$y_{k}^{i}=I_{k}^{i}\cdot h_{k}^{i}\left(x_{k}\right)+v_{k}^{i},\;i=1,2,\ldots ,L,$$

where ${\overset{˘}{x}}_{k}={\left[x_{k}^{T}\;I_{k}^{T}\right]}^{T}$, $W_{k}={\left[w_{k}^{T}\;{\tilde{w}}_{k}^{1^{T}},\ldots ,{\tilde{w}}_{k}^{L^{T}}\right]}^{T}$, $F_{k}\left(X_{k}\right)={\left[f_{k}^{T}\left(x_{k}\right)\;0\right]}^{T}$, $I_{k}={\left[I_{k}^{1},\ldots ,I_{k}^{L}\right]}^{T}$, $w_{k}∼N\left(0,Q_{k}\right)$. ${\tilde{w}}_{k}^{i}∼B\left(1,p_{k}^{i}\right)$, which means a Bernoulli distribution with probability parameter $p_{k}^{i}$, if $P\left({\tilde{w}}_{k}^{i}=1\right)=p_{k}^{i}$ and $P\left({\tilde{w}}_{k}^{i}=0\right)=1-p_{k}^{i}$. The process noise is independent of the uncertainty. Then, we assume $w_{k}$ and ${\tilde{w}}_{k}^{i}$, i=1,2,…,L are mutually independent.

Since the PCRB is an important criterion of sensor selection, we drive two PCRBs of the uncertain dynamic systems (1), (2), (6) and (7) in Section 3 and Section 4, respectively. The former is accurate, but it is difficult to be computed. Thus, the latter is derived by introduced some hidden state variables, which avoids the complex integral and can reduce the computation burden. Finally, based on the second PCRB, we hope to obtain the analytically optimal solution of the sensor selection problem, so that it can be applied to the large-scale sensor selection problem for the uncertain dynamic systems.

## 3. The Posterior Cramér–Rao Bound with Uncertain Observations

In this section, we mainly discuss two methods to calculate the PCRB of multiple sensors. The first method is based on the nonlinear dynamic system with uncertain observations (1) and (2) and Gaussian mixture model [5,13,15,32]. The other approach is based on the nonlinear dynamic system (6) and (7) motivated by the EM algorithm [33].

Let θ be a *r*-dimensional estimated random parameter, z represents a vector of measured data, let p(z,θ) be the joint probability density of the pair (z,θ), and let g(z) be a function of z, which is an estimate of θ. Let Δ and ∇ be operators of the first and second-order partial derivatives, respectively,

$$\begin{array}{cc} \nabla_{\eta } & =[\frac{\partial }{\partial \eta_{1}},\ldots ,\frac{\partial }{\partial \eta_{L}}],\\ \Delta_{\eta }^{\xi } & =\nabla_{\eta }\nabla_{\xi }^{T}. \end{array}$$

The PCRB on the estimate error has the form

(8) $$\begin{array}{c} P=E\left\{\left[g\left(z\right)-\theta \right]{\left[g\left(z\right)-\theta \right]}^{T}\right\}\geq J^{-1}, \end{array}$$

where $J=E\left\{-\Delta_{\Theta }^{\Theta }\log p_{z,\theta }\left(Z,\Theta \right)\right\}$ is the (Fisher) information matrix denoted by Van Trees [8]. For example, if the posterior distribution of θ conditioned on z is Gaussian with mean ${\bar{\theta }}_{z}$ and a covariance matric $\Sigma_{z}$. Then, the information matrix (8) reads $J=E\{\Sigma_{z}^{-1}\}$.

Assume now that the parameter θ is decomposed into two parts as $\theta ={\left[\theta_{\alpha }^{T},\theta_{\beta }^{T}\right]}^{T}$, and the information matrix J is correspondingly divided into blocks

$$\begin{array}{c} J=\left[\begin{array}{cc} J_{\alpha \alpha } & J_{\alpha \beta }\\ J_{\beta \alpha } & J_{\beta \beta } \end{array}\right]. \end{array}$$

Then, it can be easily shown that the covariance of estimation of $\theta_{\beta }$ is lower bounded by the right-lower block of $J^{-1}$, i.e.,

$$\begin{array}{ccc} P_{\beta } & = & E\{\left[g_{\beta }\left(x\right)-\theta_{\beta }\right]{\left[g_{\beta }\left(x\right)-\theta_{\beta }\right]}^{T}\}\\ & \geq & {\left[J_{\beta \beta }-J_{\beta \alpha }J_{\alpha \alpha }^{-1}J_{\alpha \beta }\right]}^{-1}, \end{array}$$

where we assume that $J_{\alpha \alpha }^{-1}$ exists. Denoted $J_{\beta }=J_{\beta \beta }-J_{\beta \alpha }J_{\alpha \alpha }^{-1}J_{\alpha \beta }$, which is called the information submatrix for β.

Now, for nonlinear dynamic systems with uncertain observations (1) and (2), the following proposition gives a method to compute the information submatrix $J_{k}$ recursively.

**Proposition** **1.** *The Fisher information submatrix $J_{k}$ for the estimating state vectors $\left\{x_{k}\right\}$ obeys the recursion:*

(9) $$\begin{array}{ccc} J_{k+1} & = & D_{k}^{22}-D_{k}^{21}{\left(J_{k}+D_{k}^{11}\right)}^{-1}D_{k}^{12}, \end{array}$$

(10) $$\begin{array}{ccc} J_{0} & = & E[-\Delta_{x_{0}}^{x_{0}}\log p\left(x_{0}\right)], \end{array}$$

*with*

(11) $$\begin{array}{ccc} D_{k}^{11} & = & E\left[\nabla_{x_{k}}f_{k}^{T}\left(x_{k}\right)\right]Q_{k}^{-1}{\left[\nabla_{x_{k}}f_{k}^{T}\left(x_{k}\right)\right]}^{T}, \end{array}$$

(12) $$\begin{array}{ccc} D_{k}^{12} & = & -E\left[\nabla_{x_{k}}f_{k}^{T}\left(x_{k}\right)\right]Q_{k}^{-1}, \end{array}$$

(13) $$\begin{array}{ccc} D_{k}^{21} & = & {\left(D_{k}^{12}\right)}^{T}, \end{array}$$

(14) $$\begin{array}{ccc} D_{k}^{22} & = & Q_{k}^{-1}+\sum_{i=1}^{L}E\left\{-\Delta_{x_{k+1}}^{x_{k+1}}\log p\left(y_{k+1}^{i}|x_{k+1}\right)\right\}, \end{array}$$

*where*

(15) $$\begin{array}{c} p\left(y_{k+1}^{i}|x_{k+1}\right)=p_{k}^{i}N\left(h_{k}^{i}\left(x_{k+1}\right),R_{k+1}^{i}\right)+\left(1-p_{k}^{i}\right)N\left(0,R_{k+1}^{i}\right). \end{array}$$

**Proof.**  Equations (1) and (2) together with $p(x_{0})$ determine the joint probability distribution of $X_{k}=\left[x_{0},x_{1},\ldots ,x_{k}\right]$ and $Y_{k}=\left[y_{0},y_{1},\ldots ,y_{k}\right]$, where $y_{k}={\left(y_{k}^{1},y_{k}^{2},\ldots ,y_{k}^{L}\right)}^{T}$,

(16) $$\begin{array}{ccc} p(X_{k},Y_{k}) & = & p\left(X_{k-1},Y_{k-1}\right)\cdot p\left(x_{k}|X_{k-1},Y_{k-1}\right)\cdot p\left(y_{k}|X_{k},Y_{k-1}\right)\\ & = & p\left(X_{k-1},Y_{k-1}\right)\cdot p\left(x_{k}|x_{k-1}\right)\cdot p\left(y_{k}|x_{k}\right)\\ & = & p\left(X_{k-1},Y_{k-1}\right)\cdot p\left(x_{k}|x_{k-1}\right)\cdot \prod_{i=1}^{L}p\left(y_{k}^{i}|x_{k}\right). \end{array}$$

The conditional probability densities $p(x_{k}|x_{k-1})$ and $p(y_{k}^{i}|x_{k})$ can be calculated by Equations (1) and (2), respectively. Denote $p_{k}=p\left(X_{k},Y_{k}\right)$, by Equation (16), we can obtain the formula about $p_{k+1}$ as follows:

(17) $$\begin{array}{c} p_{k+1}=p_{k}\cdot p\left(x_{k+1}|x_{k}\right)\cdot \prod_{i=1}^{L}p\left(y_{k+1}^{i}|x_{k+1}\right). \end{array}$$

Therefore,

(18) $$\begin{array}{c} \log p_{k+1}=\log p_{k}+\log p\left(x_{k+1}|x_{k}\right)+\sum_{i=1}^{L}\log p\left(y_{k+1}^{i}|x_{k+1}\right). \end{array}$$

If we divide $X_{k}$ into $X_{k}={\left[X_{k-1}^{T},x_{k}^{T}\right]}^{T}$, then

(19) $$\begin{array}{c} J\left(X_{k}\right)=E\left\{-\Delta_{X_{k}}^{X_{k}}\log p_{k}\right\}=\left[\begin{array}{cc} E\{-\Delta_{X_{k-1}}^{X_{k-1}}\log p_{k}\} & E\{-\Delta_{X_{k-1}}^{x_{k}}\log p_{k}\}\\ E\{-\Delta_{x_{k}}^{X_{k-1}}\log p_{k}\} & E\{-\Delta_{x_{k}}^{x_{k}}\log p_{k}\} \end{array}\right]≜\left[\begin{array}{cc} A_{k} & B_{k}\\ B_{k}^{T} & C_{k} \end{array}\right]. \end{array}$$

The information submatrix $J_{k}$ for $x_{k}$ can be obtained as follows:

(20) $$\begin{array}{c} J_{k}=C_{k}-B_{k}^{T}A_{k}^{-1}B_{k}. \end{array}$$

Moreover, let $X_{k+1}={\left[X_{k-1}^{T},x_{k}^{T},x_{k+1}^{T}\right]}^{T}$, then the posterior information matrix for $X_{k+1}$ can be written as the following block form by Equation (18),

(21) $$\begin{array}{c} J\left(X_{k+1}\right)=\left[\begin{array}{ccc} A_{k} & B_{k} & 0\\ B_{k}^{T} & C_{k}+D_{k}^{11} & D_{k}^{12}\\ 0 & D_{k}^{21} & D_{k}^{22} \end{array}\right], \end{array}$$

where 0 stands for zero blocks of appropriate dimensions, and $D_{k}^{11},D_{k}^{12},D_{k}^{22}$ are calculated as follows:

$$\begin{array}{ccc} D_{k}^{11} & = & E\{-\Delta_{x_{k}}^{x_{k}}\log p\left(x_{k+1}|x_{k}\right)\},\\ D_{k}^{12} & = & E\left\{-\Delta_{x_{k}}^{x_{k+1}}\log p\left(x_{k+1}|x_{k}\right)\right\}={\left(D_{k}^{21}\right)}^{T},\\ D_{k}^{22} & = & \sum_{i=1}^{L}E\left\{-\Delta_{x_{k+1}}^{x_{k+1}}\log p\left(y_{k+1}^{i}|x_{k+1}\right)\right\}. \end{array}$$

Then, the information submatrix $J_{k+1}$ for $x_{k+1}$ can be computed as

(22) $$\begin{array}{ccc} J_{k+1} & = & D_{k}^{22}-\left[\begin{array}{cc} 0 & D_{k}^{21} \end{array}\right]{\left[\begin{array}{cc} A_{k} & B_{k}\\ B_{k}^{T} & C_{k}+D_{k}^{11} \end{array}\right]}^{-1}\left[\begin{array}{c} 0\\ D_{k}^{12} \end{array}\right]\\ & = & D_{k}^{22}-D_{k}^{21}{\left[C_{k}+D_{k}^{11}-B_{k}^{T}A_{k}^{-1}B_{k}\right]}^{-1}D_{k}^{12}. \end{array}$$

Based on the definition of $J_{k}$ in (20), we can obtain the desired recursion (9). Since the state noise and the measurement noise are Gaussian with zero mean and invertible covariance matrices $Q_{k}$ and $R_{k}^{i}$, i=1,…,L, respectively. Moreover, the dynamic systems have the uncertain observations. From these assumptions and Equation (3), it follows that

$$\begin{array}{c} -\log p\left(x_{k+1}|x_{k}\right)=c_{1}+\frac{1}{2}{\left[x_{k+1}-f_{k}\left(x_{k}\right)\right]}^{T}Q_{k}^{-1}\left[x_{k+1}-f_{k}\left(x_{k}\right)\right]\\ -\log p\left(y_{k+1}^{i}|x_{k+1}\right)=-\log\left(p_{k}N\left(h_{k+1}^{i}\left(x_{k+1}\right),R_{k+1}^{i}\right)+\left(1-p_{k}\right)N\left(0,R_{k+1}^{i}\right)\right), \end{array}$$

where $c_{1}$ is a constant. Therefore, $D_{k}^{11},D_{k}^{12},D_{k}^{22}$ can be simplified to (11)–(14). ☐

From Equations (14) and (15), we see that the appearance of the summation inside of the logarithm, and the computation of $D_{22}^{k}$ is related to the joint probability density function of the sensor measurements $y_{k+1}$ and the target state $x_{k+1}$, then $D_{22}^{k}$ is not easy to calculate. These reasons motivate us to study another approach to derive the PCRB.

Based on the equivalence between the systems (1)–(2) and (6)–(7), we can derive PCRB for the dynamic systems (6) and (7) by introducing a hidden variable $I_{k}$, and the new PCRB may be easier to compute. Since the second derivation for the discrete augmented variable $I_{k}$ do not exist, then we bring in a continuous random variable ${\tilde{I}}_{k}$ to approximate the 0–1 variable $I_{k}$. The augmented state vector ${\overset{˘}{x}}_{k}={\left[x_{k},I_{k}\right]}^{T}$ has changed into ${\tilde{x}}_{k}={\left[x_{k},{\tilde{I}}_{k}\right]}^{T}$. Therefore, the new system can be expressed as follows:

(23) $$\begin{array}{ccc} x_{k} & = & f_{k}\left(x_{k-1}\right)+w_{k}, \end{array}$$

(24) $$\begin{array}{ccc} {\tilde{I}}_{k}^{i} & = & 0\cdot {\tilde{I}}_{k-1}^{i}+{\tilde{w}}_{k}, \end{array}$$

(25) $$\begin{array}{ccc} y_{k}^{i} & = & {\tilde{I}}_{k}^{i}\cdot h_{k}^{i}\left(x_{k}\right)+v_{k}^{i},i=1,2,\ldots ,L. \end{array}$$

**Lemma** **1** ([21]). *If $I_{k}^{i}∼B\left(1,p_{k}^{i}\right)$ and ${\tilde{I}}_{k}^{i}∼p_{k}^{i}N\left(1,\sigma^{2}\right)+\left(1-p_{k}^{i}\right)N\left(0,\sigma^{2}\right)$, i=1,…,L, then the limit of ${\tilde{I}}_{k}$ is the state variable $I_{k}$ when σ→0, i.e., $\lim_{\sigma \to 0}{\tilde{I}}_{k}=I_{k}$.*

Let ${\tilde{J}}_{k}$ represents the PCRB about the approximated augment vector ${\tilde{x}}_{k}$ of systems (23)–(25), respectively. Then, we can easily get the following conclusion:

**Lemma** **2** ([21]). *Assume that ${\tilde{I}}_{k}^{i}∼p_{k}^{i}N\left(1,\sigma^{2}\right)+\left(1-p_{k}^{i}\right)N\left(0,\sigma^{2}\right)$, i=1,…,L, then $P_{k}\left({\overset{˘}{x}}_{k}\right)\geq \lim_{\sigma \to 0}{\tilde{J}}_{k}^{-1}=\left[\begin{array}{cc} {\bar{J}}_{k}^{-1} & 0\\ 0 & 0 \end{array}\right]$, where $P_{k}\left({\overset{˘}{x}}_{k}\right)$ denotes the estimation error covariance matrix about the vector ${\overset{˘}{x}}_{k}$ and ${\bar{J}}_{k}$ denotes the Fisher information submatrix about the vector $x_{k}$.*

Based on Lemmas 1 and 2, for nonlinear dynamic system with the uncertain observations (6) and (7), it is easy to see that ${\bar{J}}_{k}^{-1}$ can also represent a CRB for the estimation error covariance matrix of vector $x_{k}$.

**Proposition** **2.** *At time k+1, the Fisher information submatrix ${\bar{J}}_{k+1}$ of $x_{k+1}$ for the multi-sensor uncertain systems (6) and (7) is computed according to the following recursion:*

(26) $$\begin{array}{ccc} {\bar{J}}_{k+1} & = & {\bar{D}}_{k}^{22}-{\bar{D}}_{k}^{21}{\left({\bar{J}}_{k}+{\bar{D}}_{k}^{11}\right)}^{-1}{\bar{D}}_{k}^{12}, \end{array}$$

(27) $$\;\begin{array}{ccc} {\bar{J}}_{0} & = & \Sigma_{0}^{-1}, \end{array}$$

*with*

(28) $$\begin{array}{ccc} {\bar{D}}_{k}^{11} & = & E\{\left[\nabla_{x_{k}}f_{k}^{T}\left(x_{k}\right)\right]Q_{k}^{-1}{\left[\nabla_{x_{k}}f_{k}^{T}\left(x_{k}\right)\right]}^{T}\}, \end{array}$$

(29) $$\begin{array}{ccc} {\bar{D}}_{k}^{12} & = & -E\left[\nabla_{x_{k}}f_{k}^{T}\left(x_{k}\right)\right]Q_{k}^{-1}, \end{array}$$

(30) $$\begin{array}{ccc} {\bar{D}}_{k}^{21} & = & {\left({\bar{D}}_{k}^{12}\right)}^{T}, \end{array}$$

(31) $$\begin{array}{ccc} {\bar{D}}_{k}^{22} & = & Q_{k}^{-1}+\sum_{i=1}^{L}p_{k+1}^{i}E\left\{{\left[\nabla_{x_{k+1}}h_{k+1}^{i}\left(x_{k+1}\right)\right]}^{T}{\left(R_{k+1}^{i}\right)}^{-1}\left[\nabla_{x_{k+1}}h_{k+1}^{i}\left(x_{k+1}\right)\right]\right\}. \end{array}$$

**Proof.**  According to Lemma 1 and the derivation of Proposition 1, the new augmented state vector ${\tilde{x}}_{k}$ has the following PCRB for systems (6) and (7):

(32) $$\begin{array}{ccc} {\tilde{J}}_{k+1} & = & {\tilde{D}}_{k}^{22}-{\tilde{D}}_{k}^{21}{\left({\tilde{J}}_{k}+{\tilde{D}}_{k}^{11}\right)}^{-1}{\tilde{D}}_{k}^{12}, \end{array}$$

where ${\tilde{D}}_{k}^{11}$, ${\tilde{D}}_{k}^{12}$, ${\tilde{D}}_{k}^{22}$ are denoted as follows:

$$\begin{array}{ccc} {\tilde{D}}_{k}^{11} & = & E_{{\tilde{x}}_{k}}\left\{-\Delta_{{\tilde{x}}_{k}}^{{\tilde{x}}_{k}}\log p\left({\tilde{x}}_{k+1}|{\tilde{x}}_{k}\right)\right\},\\ {\tilde{D}}_{k}^{12} & = & E_{{\tilde{x}}_{k}}\left\{-\Delta_{{\tilde{x}}_{k}}^{{\tilde{x}}_{k+1}}\log p\left({\tilde{x}}_{k+1}|{\tilde{x}}_{k}\right)\right\},\\ {\tilde{D}}_{k}^{21} & = & {\left({\tilde{D}}_{k}^{12}\right)}^{T},\\ {\tilde{D}}_{k}^{22} & = & E_{{\tilde{x}}_{k+1}}\left\{-\Delta_{{\tilde{x}}_{k+1}}^{{\tilde{x}}_{k+1}}\log p\left({\tilde{x}}_{k+1}|{\tilde{x}}_{k}\right)\right\}+\sum_{i=1}^{L}E_{{\tilde{x}}_{k+1}}\left\{-\Delta_{{\tilde{x}}_{k+1}}^{{\tilde{x}}_{k+1}}\log p\left(y_{k+1}^{i}|{\tilde{x}}_{k+1}\right)\right\}. \end{array}$$

In order to obtain the lower bound for $x_{k}$, it is necessary for us to calculate the following probability densities, according to Equations (6) and (7),

(33) $$\begin{array}{c} -\log p\left({\tilde{x}}_{k+1}|{\tilde{x}}_{k}\right)=-\log p\left(x_{k+1}|x_{k}\right)\cdot p\left({\tilde{I}}_{k+1}\right)\\ =c_{3}+\frac{1}{2}{\left[x_{k+1}-f_{k}\left(x_{k}\right)\right]}^{T}Q_{k}^{-1}\left[x_{k+1}-f_{k}\left(x_{k}\right)\right]-\log p\left({\tilde{I}}_{k+1}\right), \end{array}$$

where $c_{3}$ is a constant, and the first equality follows from the independence and the second follows from (2). The another probability density is as follows:

(34) $$\begin{array}{c} -\log p\left(y_{k+1}^{i}|{\tilde{x}}_{k+1}\right)=c_{4}+\frac{1}{2}{\left[y_{k+1}^{i}-{\tilde{I}}_{k+1}^{i}h_{k+1}^{i}\left(x_{k+1}\right)\right]}^{T}{\left(R_{k+1}^{i}\right)}^{-1}\left[y_{k+1}^{i}-{\tilde{I}}_{k+1}^{i}h_{k+1}^{i}\left(x_{k+1}\right)\right], \end{array}$$

where $c_{4}$ is a constant. Since ${\tilde{x}}_{k}={\left[x_{k},{\tilde{I}}_{k}\right]}^{T}$, we use Equations (33)–(34) and Lemma 1, and the suitable partitioned expressions for ${\tilde{D}}_{k}^{11}$, ${\tilde{D}}_{k}^{12}$, ${\tilde{D}}_{k}^{22}$ are obtained:

(35) $$\begin{array}{ccc} {\tilde{D}}_{k}^{11} & = & \left[\begin{array}{cc} {\bar{D}}_{k}^{11} & 0\\ 0 & 0 \end{array}\right], \end{array}$$

(36) $$\begin{array}{ccc} {\tilde{D}}_{k}^{12} & = & \left[\begin{array}{cc} {\bar{D}}_{k}^{12} & 0\\ 0 & 0 \end{array}\right], \end{array}$$

(37) $$\begin{array}{ccc} {\tilde{D}}_{k}^{22} & = & \left[\begin{array}{cc} {\bar{D}}_{k}^{22} & C_{12}\\ C_{21} & C_{22} \end{array}\right], \end{array}$$

where ${\bar{D}}_{k}^{11}$, ${\bar{D}}_{k}^{12}$ are denoted in (28) and (29), while $C_{12},C_{21}$ and $C_{22}$ are calculated as follows:

$$\begin{array}{ccc} C_{12} & = & E_{{\tilde{x}}_{k+1}}\left\{-\Delta_{x_{k+1}}^{{\tilde{I}}_{k+1}}\log p\left({\tilde{x}}_{k+1}|{\tilde{x}}_{k}\right)\right\}+\sum_{i=1}^{L}E_{{\tilde{x}}_{k+1}}\left\{-\Delta_{x_{k+1}}^{{\tilde{I}}_{k+1}^{i}}\log p\left(y_{k+1}^{i}|{\tilde{x}}_{k+1}\right)\right\}\\ & = & -\sum_{i=1}^{L}E_{{\tilde{x}}_{k+1}}\left\{{\left[\nabla_{x_{k+1}}h_{k+1}^{i}\left(x_{k+1}\right)\right]}^{T}{\left(R_{k+1}^{i}\right)}^{-1}y_{k+1}^{i}\right\}\\ & & +2\sum_{i=1}^{L}E_{{\tilde{x}}_{k+1}}\left\{{\tilde{I}}_{k+1}^{i}h_{k+1}^{i}\left(x_{k+1}\right){\left(R_{k+1}^{i}\right)}^{-1}\left[\nabla_{x_{k+1}}h_{k+1}^{i}\left(x_{k+1}\right)\right]\right\}\\ & = & {\left(C_{21}\right)}^{T}, \end{array}$$

$$\begin{array}{ccc} C_{22} & = & \sum_{i=1}^{L}E_{{\tilde{x}}_{k+1}}\left\{-\Delta_{{\tilde{I}}_{k+1}^{i}}^{{\tilde{I}}_{k+1}^{i}}\log p\left(y_{k+1}^{i}|{\tilde{x}}_{k+1}\right)\right\}+\sum_{i=1}^{L}E_{{\tilde{x}}_{k+1}}\left\{-\Delta_{{\tilde{I}}_{k+1}^{i}}^{{\tilde{I}}_{k+1}^{i}}\log p\left({\tilde{I}}_{k+1}^{i}\right)\right\}\\ & = & \sum_{i=1}^{L}E_{{\tilde{x}}_{k+1}}\left\{{\left(h_{k+1}^{i}\left(x_{k+1}\right)\right)}^{T}{\left(R_{k+1}^{i}\right)}^{-1}\left(h_{k+1}^{i}\left(x_{k+1}\right)\right)\right\}+\sum_{i=1}^{L}E_{{\tilde{x}}_{k+1}}\left\{-\Delta_{{\tilde{I}}_{k+1}^{i}}^{{\tilde{I}}_{k+1}^{i}}\log p\left({\tilde{I}}_{k+1}^{i}\right)\right\}. \end{array}$$

If we divide ${\tilde{J}}_{k}$ as the following block matrix

(38) $$\begin{array}{c} {\tilde{J}}_{k}=\left[\begin{array}{cc} {\tilde{J}}_{k}^{11} & {\tilde{J}}_{k}^{12}\\ {\tilde{J}}_{k}^{21} & {\tilde{J}}_{k}^{22} \end{array}\right], \end{array}$$

then according to (32) and (35)–(37), the value of ${\tilde{J}}_{k+1}$ is

(39) $$\begin{array}{c} {\tilde{J}}_{k+1}=\left[\begin{array}{cc} {\bar{D}}_{k}^{22}+{\bar{D}}_{k}^{21}{\left({\bar{D}}_{k}^{11}+{\tilde{J}}_{k}^{11}-{\tilde{J}}_{k}^{12}{\left({\tilde{J}}_{k}^{22}\right)}^{-1}{\tilde{J}}_{k}^{21}\right)}^{-1}{\bar{D}}_{k}^{12} & C_{12}\\ C_{21} & C_{22} \end{array}\right]. \end{array}$$

Since the matrix $C_{22}$ is the function of σ, it is shown in [21] that

(40) $$\begin{array}{c} \lim_{\sigma \to 0}{\tilde{J}}_{k+1}^{-1}=\left[\begin{array}{cc} {\left({\bar{D}}_{k}^{22}+{\bar{D}}_{k}^{21}{\left({\bar{D}}_{k}^{11}+{\bar{J}}_{k}^{11}\right)}^{-1}{\bar{D}}_{k}^{12}\right)}^{-1} & 0\\ 0 & 0 \end{array}\right]. \end{array}$$

Using Lemma 1, we can obtain (26), and the matrix ${\bar{D}}_{k}^{22}$ can be computed as

$$\begin{array}{ccc} {\bar{D}}_{k}^{22} & = & \sum_{i=1}^{L}E_{{\tilde{x}}_{k+1}}\left\{I_{k+1}^{i}\nabla_{x_{k+1}}{\left(h_{k+1}^{i}\left(x_{k+1}\right)\right)}^{T}{\left(R_{k+1}^{i}\right)}^{-1}\nabla_{x_{k+1}}\left(h_{k+1}^{i}\left(x_{k+1}\right)\right)I_{k+1}^{i}\right\}+Q_{k}^{-1}\\ & = & Q_{k}^{-1}+\sum_{i=1}^{L}p_{k+1}^{i}E_{x_{k+1}}\left\{{\left[\nabla_{x_{k+1}}h_{k+1}^{i}\left(x_{k+1}\right)\right]}^{T}{\left(R_{k+1}^{i}\right)}^{-1}\left[\nabla_{x_{k+1}}h_{k+1}^{i}\left(x_{k+1}\right)\right]\right\}. \end{array}$$

 ☐

**Remark** **1.** Note that PCRBs derived in Propositions 1 and 2 have different forms. The first one is optimal. The second one is only approximately optimal with less computational burden. Since it may be approximated from above or below, which one is lower cannot be judged. The simulation in Section 6 shows that they are almost equal and the computational complexity of the approximate bound is less than that of the accurate bound.

**Remark** **2.** In the case of p=1 and L=1, the multi-sensor dynamic systems (1) and (2) has the certain observations. Obviously, the PCRB derived by the method in [13] is consistent with that derived in Proposition 2.

## 4. Sensor Selection with Uncertain Observations

In large sensor networks, it is an important problem to manage the communication resources efficiently. The calculation of PCRB by Proposition 1 needs to use the joint probability density function of the sensor measurements and the target state, which leads to the computational burden being heavy, so that it is detrimental to be used as a measure criteria of the sensor selection. In this section, we consider the problem of sensor selection by Proposition 2.

For the nonlinear dynamic system at time *k*, assume that *s* sensors will be selected from *L* sensors by maximizing the Fisher information matrix, then they will send their measurements or local estimates to the fusion center. Finally, the fusion center makes the estimates for the state. In order to select the optimal *s* sensors, we need to introduce a selection vector $s_{k}={\left[s_{k}^{1},\ldots ,s_{k}^{L}\right]}^{T}\in {\left\{0,1\right\}}^{L}$. If the *i*th sensor is selected, let $s_{k}^{i}=1$; otherwise, $s_{k}^{i}=0$, i=1,…,L. According to the derivation of the Fisher information matrix in Section 3, the selection vector modifies the log conditional probability density $logp(y_{k}|x_{k})$ as [34]

(41) $$\begin{array}{c} log\prod_{i=1}^{L}{\left(p\left(y_{k}^{i}|x_{k}\right)\right)}^{s_{k}^{i}}=\sum_{i=1}^{L}s_{k}^{i}logp\left(y_{k}^{i}|x_{k}\right). \end{array}$$

In fact, the selected variable $s_{k}$ only has an effect on ${\bar{D}}_{k}^{22}$ of Proposition 2. Then, ${\bar{D}}_{k}^{22}$ can be written as

(42) $$\begin{array}{ccc} {\bar{D}}_{k}^{22} & = & Q_{k}^{-1}+\sum_{i=1}^{L}s_{k+1}^{i}p_{k+1}^{i}E\left\{{\left[\nabla_{x_{k+1}}h_{k+1}^{i}\left(x_{k+1}\right)\right]}^{T}{\left(R_{k+1}^{i}\right)}^{-1}\left[\nabla_{x_{k+1}}h_{k+1}^{i}\left(x_{k+1}\right)\right]\right\}. \end{array}$$

Therefore, the information matrix of $x_{k+1}$ is the function of the selected variable $s_{k}$. Now, the sensor selection problem can be expressed as the following optimization problem:

(43) $$\begin{array}{cc} & \max_{s_{k+1}}\;tr\left({\bar{J}}_{k+1}\left(s_{k+1}\right)\right), \end{array}$$

(44) $$\begin{array}{c} \;s.t.\;\sum_{i=1}^{L}s_{k+1}^{i}=s, \end{array}$$

(45) $$\;\begin{array}{cc} & s_{k+1}^{i}\in \left\{0,1\right\},\;i=1,\ldots ,L, \end{array}$$

where “tr” means “trace”, which is the sum of squares of semiaxes lengths of the Fisher information matrix. “s.t”. means “subjected to”.

**Remark** **3.** In fact, the objective function in (43) should be matrix ${\bar{J}}_{k+1}\left(s_{k+1}\right)$. Then, the problem (43)–(45) is a matrix optimization problem, which is considered in the sense that if $s_{k+1}^{*}$ is an optimal solution. Then, for an arbitrary feasible solution $s_{k+1}$, we have ${\bar{J}}_{k+1}\left(s_{k+1}\right)⪰{\bar{J}}_{k+1}\left(s_{k+1}^{*}\right)$, i.e., ${\bar{J}}_{k+1}\left(s_{k+1}\right)-{\bar{J}}_{k+1}\left(s_{k+1}^{*}\right)$ is a positive semidefinite matrix. There are two reasons to choose trace function as the objective function. First, it is a linear function, which helps us to easily derive the optimal solution. Second, some researchers [26,27,28] have proved that it has many advantages to apply to sensor selection, such as, if the primal matrix optimization problem has an optimal solution and ${\bar{D}}_{k}^{12}$ in (29) is invertible, then the matrix optimization problem for sensor selection can be equivalently transformed to this convex optimization problem (43)–(45).

Let the information measure corresponding to the *i*-th sensor at k+1-th time be denoted as

(46) $$\begin{array}{ccc} b_{k+1}^{i} & = & p_{k+1}^{i}tr\left(E\left\{{\left[\nabla_{x_{k+1}}h_{k+1}^{i}\left(x_{k+1}\right)\right]}^{T}{\left(R_{k+1}^{i}\right)}^{-1}\left[\nabla_{x_{k+1}}h_{k+1}^{i}\left(x_{k+1}\right)\right]\right\}\right),i=1,\ldots ,L. \end{array}$$

Let $\left\{b_{k+1}^{r_{1}},\ldots ,b_{k+1}^{r_{L}}\right\}$ denote $\left\{b_{k+1}^{1},\ldots ,b_{k+1}^{L}\right\}$ as rearrangement with descending order, i.e., $b_{k+1}^{r_{1}}\geq \ldots \geq b_{k+1}^{r_{L}}$. The optimal solution of the optimization problem (43)–(45) can be obtained by the following proposition.

**Proposition** **3.** *For multisensor nonlinear dynamic system with the uncertain observations (1) and (2), the optimal sensor selection scheme for the problem (43)–(45) is $s_{k+1}^{r_{1}}=\ldots =s_{k+1}^{r_{s}}=1$ and $s_{k+1}^{r_{s+1}}=\ldots =s_{k+1}^{r_{L}}=0$.*

**Proof.**  Since ${\bar{D}}_{k}^{11}$ and ${\bar{D}}_{k}^{12}$ are not related to $s_{k}$, based on Proposition 2, the optimization problem (43)–(45) can be equivalent to

(47) $$\begin{array}{cc} & \max_{s_{k+1}}\;\sum_{i=1}^{L}b_{k+1}^{i}s_{k+1}^{i}, \end{array}$$

(48) $$\begin{array}{c} s.t.\;\sum_{i=1}^{L}s_{k+1}^{i}=s, \end{array}$$

(49) $$\;\begin{array}{cc} & s_{k+1}^{i}\in \left\{0,1\right\},\;i=1,\ldots ,L, \end{array}$$

where $b_{k+1}^{i}$ is denoted by (46). According to $b_{k+1}^{r_{1}}\geq \ldots \geq b_{k+1}^{r_{L}}$, and $s_{k+1}^{i}$, i=1,…,L needs to satisfy (48) and (49), and we have

$$\begin{array}{c} \sum_{i=1}^{L}b_{k+1}^{i}s_{k+1}^{i}\leq \sum_{i=1}^{s}b_{k+1}^{r_{i}}. \end{array}$$

The equality holds with $s_{k+1}^{r_{1}}=\ldots =s_{k+1}^{r_{s}}=1$ and $s_{k+1}^{r_{s+1}}=\ldots =s_{k+1}^{r_{L}}=0$. Thus, the optimal solution is got. ☐

## 5. Simulation

In this section, we provide two examples to compare the different PCRB by Proposition 1 with Proposition 2, and select the optimal sensors by Proposition 3.

*Example 1*: Consider an uncertain nonlinear dynamic system for the mobile robot. At time *k*, the mobile robot pose is described with thestate vector $x_{k}=\left[x_{k}\;y_{k}\;\theta_{k}\right]$, where $x_{k}$ and $y_{k}$ are the coordinates on a 2D plane relative to an external coordinate frame, and $\theta_{k}$ is the heading angle. We use the control commands $u_{k}=\left[\Delta d_{k},\Delta \theta_{k}\right]$ to determine the motion of the mobile robot, where $\Delta d_{k}$ is the incremental distance robot (in meters) and $\Delta \theta_{k}$ is the incremental change in heading angle (in degrees). The robot motion can be described as follows [35]:

$$\begin{array}{ccc} f_{1,k} & = & x_{k-1}+\Delta d_{k}cos\left(\theta_{k-1}+\frac{1}{2}\Delta \theta_{k}\right),\\ f_{2,k} & = & y_{k-1}+\Delta d_{k}sin\left(\theta_{k-1}+\frac{1}{2}\Delta \theta_{k}\right),\\ f_{3,k} & = & \theta_{k-1}+\Delta \theta_{k}, \end{array}$$

where $\Delta d_{k}=5,\Delta \theta_{k}=5$. The state equation is defined as $f_{k}={\left[f_{1,k},\;f_{2,k},\;f_{3,k}\right]}^{T}$, and then the state model is

(50) $$\begin{array}{c} x_{k}=f_{k}\left(x_{k-1},u_{k}\right)+w_{k}. \end{array}$$

The measurement equation is

(51) $$\begin{array}{ccc} y_{k}^{i} & = & \left\{\begin{array}{cc} h^{i}\left(x_{k}\right)+v_{k}^{j}, & \mathrm{with}\;\mathrm{probability}\;p_{k}^{i}\;,\\ v_{k}^{j} & \mathrm{with}\;\mathrm{probability}\;1-p_{k}^{i}\;, \end{array}\right.\\ & & for\;i=1,\ldots ,L,\;p_{k}^{i}=0.8, \end{array}$$

where

$$\begin{array}{ccc} h^{i}\left(x_{k}\right) & = & \left[\begin{array}{c} \sqrt{{\left(x_{k}\left(1\right)-z_{k}^{i}\left(1\right)\right)}^{2}+{\left(x_{k}\left(2\right)-z_{k}^{i}\left(2\right)\right)}^{2}}\\ arctan\left(\frac{x_{k}\left(2\right)-z_{k}^{i}\left(2\right)}{x_{k}\left(1\right)-z_{k}^{i}\left(1\right)}\right) \end{array}\right]. \end{array}$$

**Figure 1:**
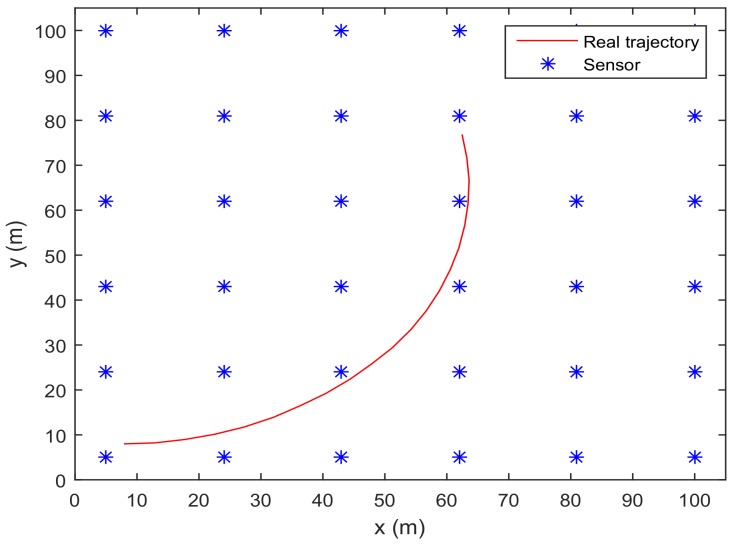
The trajectory of the mobile robot and the location of the *L* sensors.

$z_{k}^{i}={\left[z_{k}^{i}\left(1\right)\;z_{k}^{i}\left(2\right)\right]}^{T}$ is the position of the *i*th sensor. In the simulation, we consider the WSN shown in Figure 1, which has L=6×6=36 sensors deployed in the area 100×100 m${}^{2}$ [5]. The noise covariances are set as $Q_{k}=diag\left(\left[0.1^{2},0.1^{2},3^{2}\right]\right)$, $R_{k}^{i}=diag\left(\left[1^{2},1^{2}\right]\right)$.

In the example, the initial state of the robot starts is [8,8,1] and the initial covariance matrix is $P_{0}=diag\left(\left[10,10,2\right]\right)$ [35]. The sampling length is assigned to flag=50. Here, the number of Monte Carlo (MC) simulation is MC=200.

The following simulation results include three parts: the first part is about the trajectory of the mobile robot and PCRB of the state estimation, the second part is about the average computation time, and the third part is about the PCRB with different sensing probability *p*.

**Figure 2:**
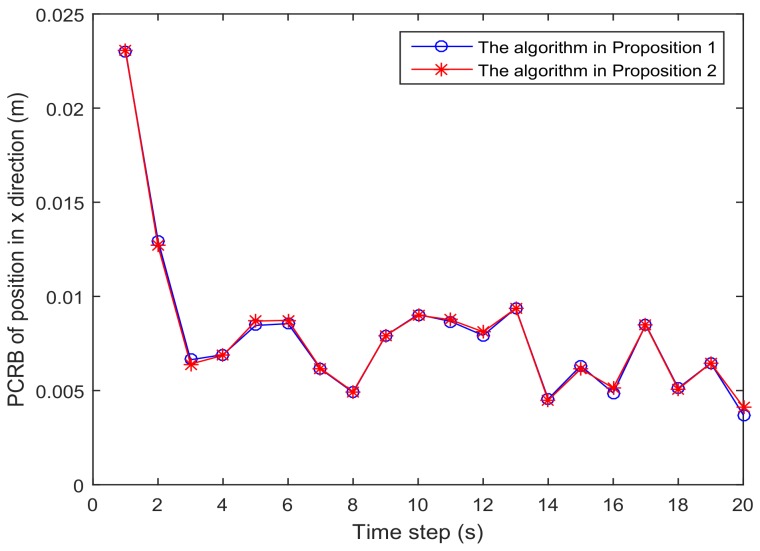
The PCRB of position in the *x*-direction is plotted as a function of time steps.

**Figure 3:**
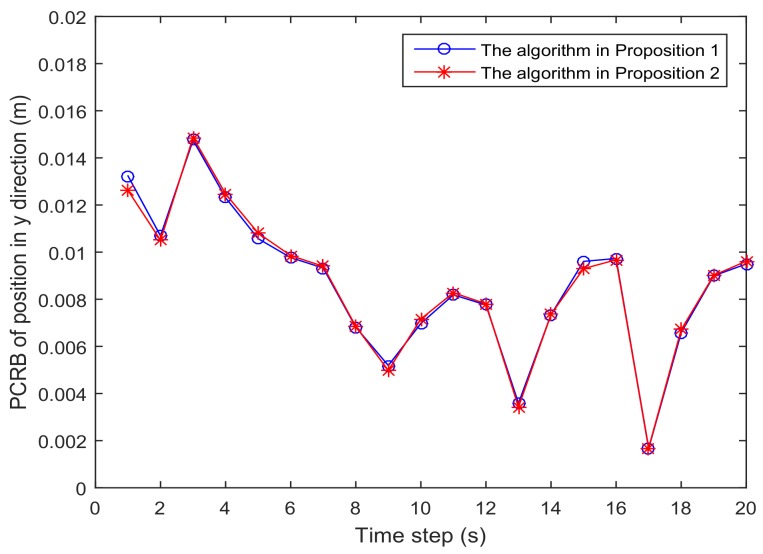
The PCRB of position in the *y*-direction is plotted as a function of time steps.

**Figure 4:**
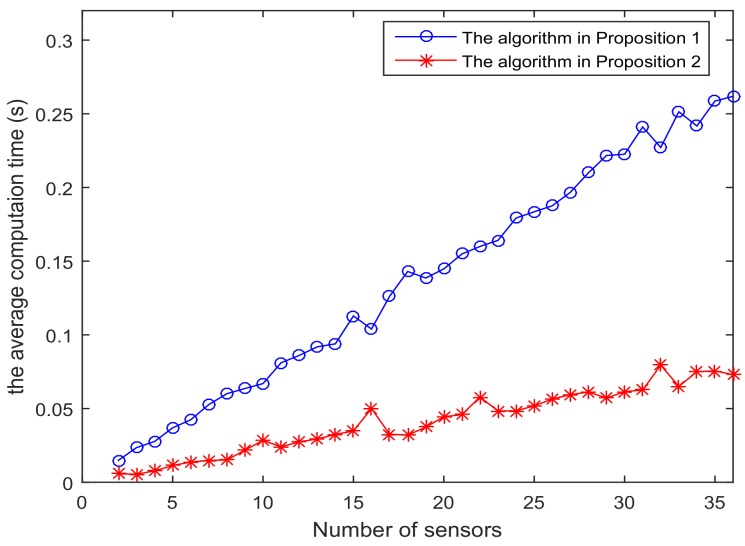
The average computation time of PCRB is plotted as function of number of sensors.

**Figure 5:**
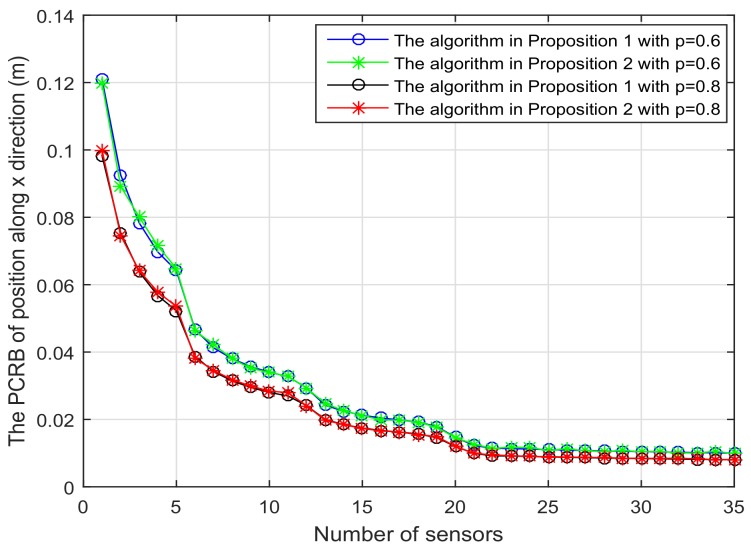
The average PCRB of 20 time steps is plotted as function of number of sensors with the different probability *p*.

- Figure 1 shows the trajectory of the mobile robot and the location of the *L* sensors. Figure 2 and Figure 3 show that the PCRB of position along the *x*- and *y*-directions based on Proposition 1 and Proposition 2, respectively. From Figure 2 and Figure 3, we can see the different PCRBs are shown to converge to the same values. However, the PCRB changes so much in the first seconds, and there are two possible reasons. First, the dynamic system is nonlinear. It may cause the algorithm to require some time to be convergent. Second, the initial variance may not be given better, such that it is far away from the convergence point.
- The average computation time of calculating PCRB based on Proposition 1 and Proposition 2 is presented in Figure 4. From Figure 4, obviously, when the number of the sensors increases, the computational complexity of Proposition 1 is much higher than that of Proposition 2, and the average computation time of PCRB by Proposition 2 increases slowly. The reason may be that the expression of PCRB based on Proposition 2 has a more concise form where the ${\bar{D}}_{k}^{22}$ is easier to compute. Thus, Proposition 2 is more suitable for the sensor selection in the large-scale sensor networks.
- In Figure 5, the average PCRB of 20 time steps is plotted as a function of number of sensors. It shows that the PCRB obtained by Proposition 1 is the same as that based on Proposition 2. The larger *p* is, the smaller the number of required sensors. The reason may be that the sensors can take more observation information, when the sensor probability *p* is larger.

*Example 2*: In order to manage the communication resources efficiently in large wireless sensor networks, we need to select some appropriate sensors. Thus, let us consider the above dynamic system (50) and (51) and the WSNs [35]. In general, if the sensors are close to the target, which may have higher sensing probabilities compared to other sensors in the WSN, then it is highly likely to select those sensors, owing to being both closer to target and higher sensing probability. Here, we consider a relatively difficult case that the sensors around the target have relatively low sensing probabilities. Then, we compare our algorithm in Proposition 3 with the recent two methods given in [5,28].

In this example, we present two cases with different number of sensors in WSN. Firstly, we consider L=36 and $s_{k}=15$. Moreover, let $p_{k}^{i}=0.1$, i=7,8,9,10,13,14,15,16,20,21,22,23,24, and the other sensing probabilities are between 0.8 and 1. Secondly, we also consider L=49 and $s_{k}=15$. Moreover, let $p_{k}^{i}=0.1$, i=14,…,18,22,…,26,30,…,34, and the other sensing probabilities are between 0.8 and 1. The following simulation results contain three parts. The first part is about the sensor selection in the application of wireless sensor network, the second part is about the mean squared error based on the selected sensors, and the third part is about the computation time.

**Figure 6:**
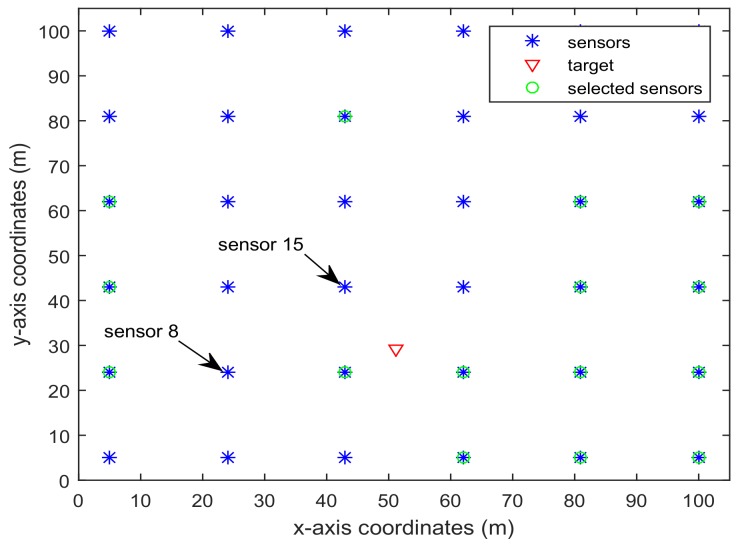
L=36 sensors placement and selected sensors $s_{k}=15$ based on the algorithm in Proposition 3.

**Figure 7:**
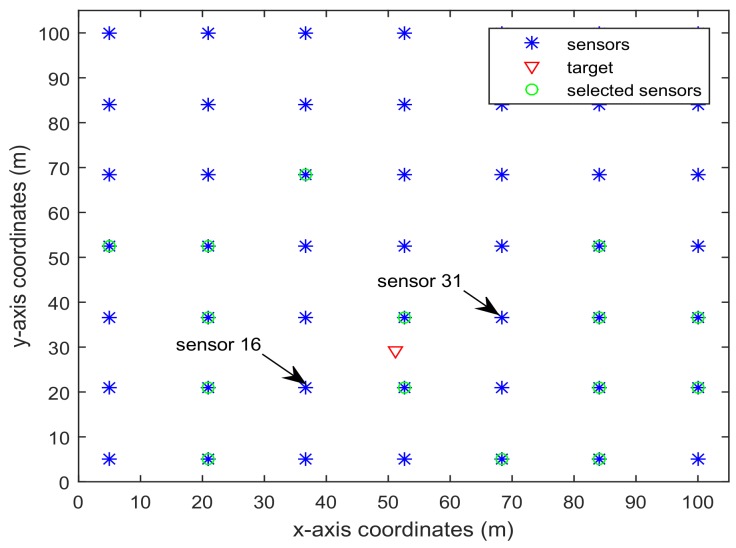
L=49 sensors placement and selected sensors $s_{k}=15$ based on the algorithm in 3.

**Figure 8:**
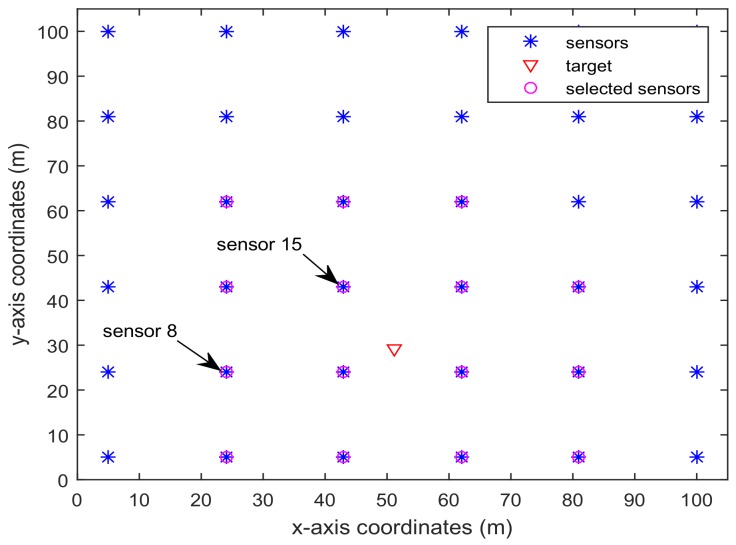
L=36 sensors placement and selected sensors $s_{k}=15$ based on the algorithm in [28].

**Figure 9:**
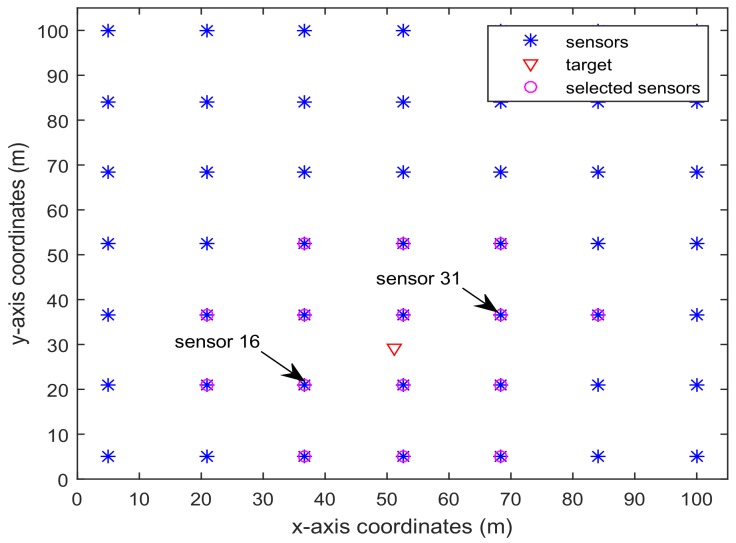
L=49 sensors placement and selected sensors $s_{k}=15$ based on the algorithm in [28].

**Figure 10:**
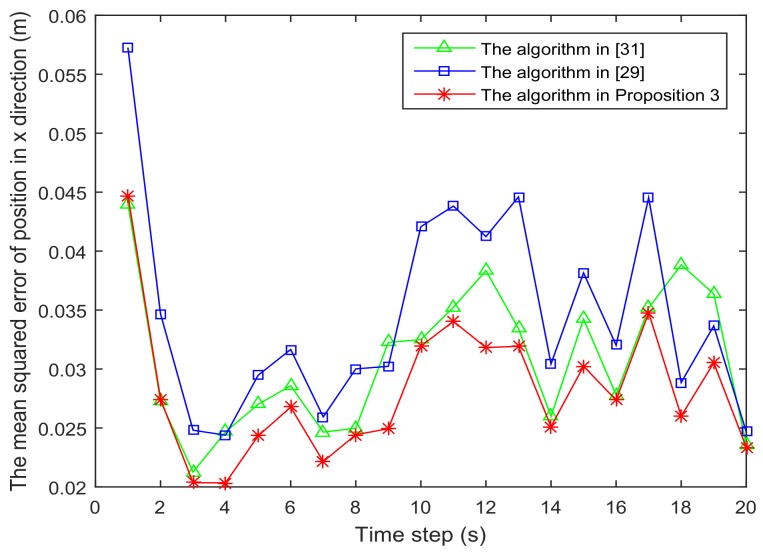
The mean squared error of position in the *x*-direction is plotted as function of time steps with L=36 sensors.

**Figure 11:**
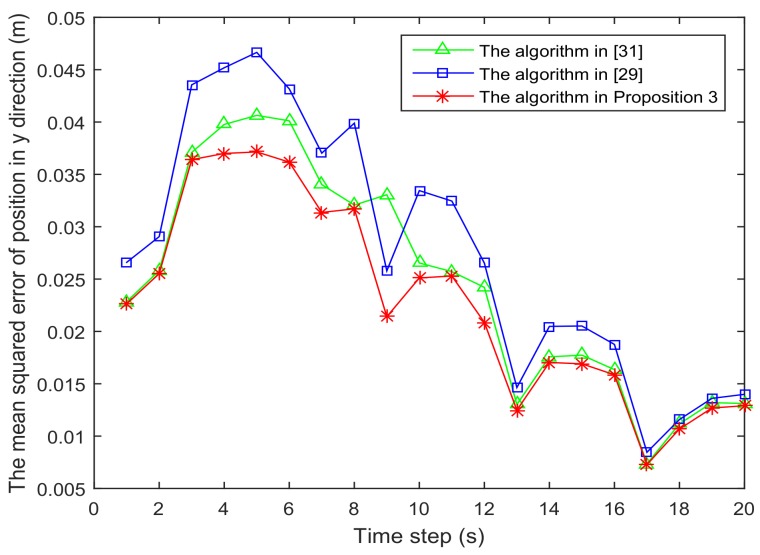
The mean squared error of position in the *y*-direction is plotted as function of time steps with L=36 sensors.

**Figure 12:**
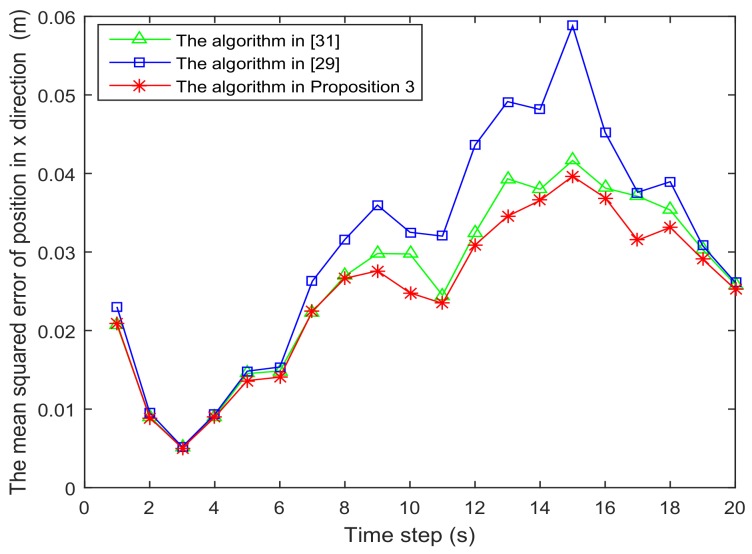
The mean squared error of position in the *x*-direction is plotted as function of time steps with L=49 sensors.

**Figure 13:**
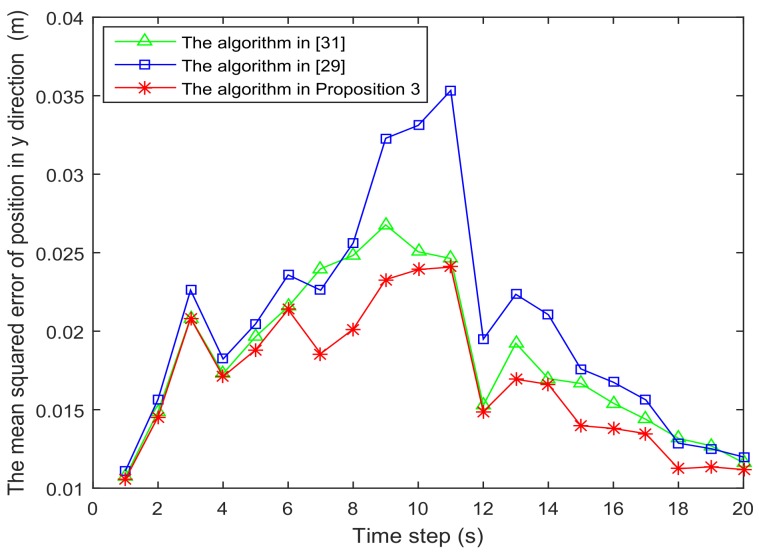
The mean squared error of position in the *y*-direction is plotted as function of time steps with L=49 sensors.

**Figure 14:**
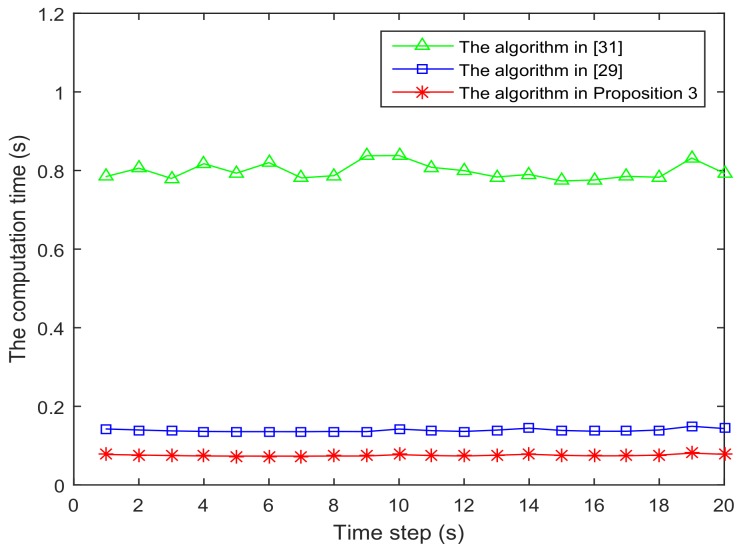
The computation time of obtaining PCRB is plotted as function of time steps with L=36 sensors.

**Figure 15:**
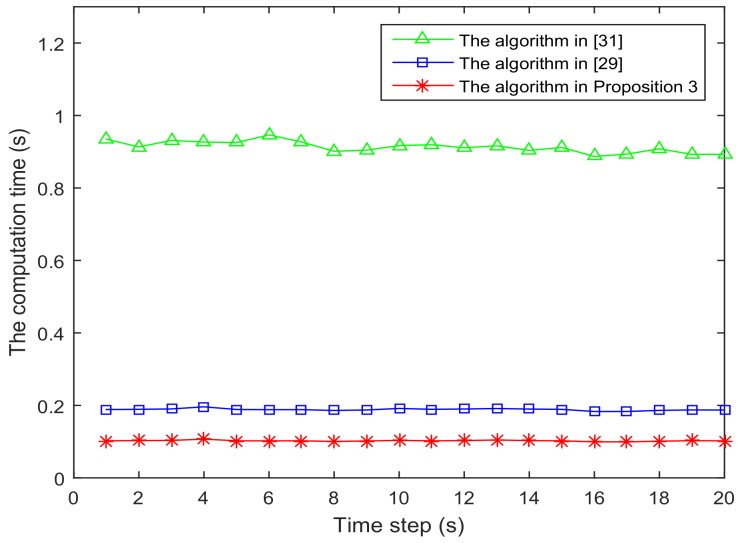
The computation time of obtaining PCRB is plotted as function of time steps with L=49 sensors.

- Figure 6 and Figure 7 present the location of L=36 and L=49 sensors, respectively. The target is showed at the time 10 s, and we use our algorithm in Proposition 3 to select the optimal $s_{k}=15$ sensors. When the uncertainty in the dynamic system is ignored, the recent method in [28] can be used to select the required sensors, and the results are shown in Figure 8 and Figure 9. Comparing Figure 6 with Figure 8, some sensors are close to the target, such as sensor 8 and sensor 15, but they are not selected in Figure 6. In Figure 8, they are selected and the only closer sensors can be selected. The reason is that the sensing probability of sensor 8 and sensor 15 is very low, and they may be not given us much useful information, although they are close to the target. Comparing Figure 7 with Figure 9, it has a similar phenomenon, such as sensor 16 and 31 not being selected in Figure 7, but they are selected in Figure 9.
- In Figure 10 and Figure 11, the mean squared errors of position in *x*- and *y*-directions are plotted for the algorithm given in Proposition 3 and the algorithms in [5,28]. It shows that our algorithm can derive the best performance. The reason is that our algorithm considers the influence of uncertain observation, and the optimal selected sensors are obtained. Although the algorithm in [5] considers the uncertain observation, it is difficult to obtain the optimal selected sensors, since it involves relaxing the variable {0,1} to the interval [0,1]. From Figure 12 and Figure 13, we can also see that the proposed method also performs best in the case of L=49, thus the performance of the new method is stable with the increase of the number of sensors.
- The computation times of obtaining PCRB are plotted in Figure 14 and Figure 15 for the three algorithms, respectively. Figure 14 and Figure 15 show that the computation time of the method in Proposition 3 is much smaller than that of the other two methods. The reason is that the method in Proposition 3 is an analytical solution. Therefore, the proposed algorithm in Proposition 3 is more suitable for the large sensor networks.

## 6. Conclusions

This paper has proposed two methods to derive the PCRB to effectively overcome the difficulties caused by uncertainty. The first method is based on the recursive formula of the Cramér–Rao bound and the Gaussian mixture model. Nevertheless, it needs to compute a complex integral based on the joint probability density function of the sensor measurements and the target state. The computational burden of this method is relatively high, especially in large sensor networks. Inspired by the idea of the expectation maximization algorithm, the second method is to introduce some 0–1 latent variables to treat the Gaussian mixture model. Since the regular condition of the posterior Cramér–Rao bound is unsatisfied for the discrete uncertain system, we use some continuous variables to approximate the discrete latent variables. Then, a new Cramér–Rao bound can be achieved by a limiting process of the Cramér–Rao bound of the continuous system. It avoids the complex integral, which can reduce the computation burden. Thus, the sensor selection problems for the nonlinear uncertain dynamic system with linear equality or inequality constraints can be efficiently solved, and the optimal solution of the sensor selection problem can be derived analytically. Thus, it can be used to deal with the sensor selection of large-scale sensor networks. Two typical numerical examples verify the effectiveness of the proposed methods.
